# Simultaneous reconstruction of bulbar urethra and anal sphincter complex: A case report on dual repair in trauma management

**DOI:** 10.1097/MD.0000000000048469

**Published:** 2026-05-08

**Authors:** Jingxing Chen, Xiao Wang, Wei Peng, Qian Liang, Ningchao Du

**Affiliations:** a Anorectal Department, The Third People’s Hospital of Longgang, Clinical Institute of Shantou University Medical College, Shenzhen, Guangdong, China; b Department of Traditional Chinese Medicine Diagnosis, Guangzhou University of Chinese Medicine, Guangzhou, Guangdong, China; c Ultrasonic Department, The Third People’s Hospital of Longgang, Clinical Institute of Shantou University Medical College, Shenzhen, Guangdong, China; d Anorectal Department, Shenzhen Second People’s Hospital (The First Affiliated Hospital of Shenzhen University), Shenzhen, Guangdong, China.

**Keywords:** anal sphincter complex, layered suture, perioperative management, urethral injury

## Abstract

**Rationale:**

Isolated urethral or anal sphincter complex injuries are rare in clinical practice, and combined traumatic injuries to both structures are extremely uncommon. The management of such complex injuries is highly challenging because it requires simultaneous reconstruction of urinary and anorectal functions.

**Patient concerns:**

A 50-year-old male patient suffered from persistent perineal bleeding and severe pain for 40 minutes after a slip-induced impalement injury to the perineum.

**Diagnoses:**

Traumatic laceration of the bulbar urethra, anal sphincter complex injury, perineal soft tissue injury, and moderate anemia.

**Interventions:**

The patient received one-stage surgery, including urethral repair, anal sphincter complex repair, perineal and anal plasty, and debridement and suture. Postoperative management included fasting, parenteral nutrition, defecation regulation, infection prevention, wound care, and anemia treatment.

**Outcomes:**

The perineal wound healed successfully. The patient recovered normal urination, fecal continence, and sexual activity. No severe complications, such as wound dehiscence, urethral stricture, or fecal incontinence, occurred.

**Lessons:**

For patients with combined traumatic injuries of the urethra and anal sphincter complex, careful preoperative evaluation, mastery of local surgical anatomy, appropriate suture materials, and precise surgical techniques are essential. Meticulous postoperative care, including dietary management, defecation assistance, and strict wound hygiene, is critical for successful functional recovery.

## 1. Background

This case underscores the importance of accurately assessing anatomical structures during the diagnosis and treatment of perineal complex injuries. Precise mucosal apposition during urethral repair is essential, while sphincter repair should involve a layered suture technique, including end-to-end suturing of the internal sphincter and overlapping suturing of the external sphincter, to minimize the risk of postoperative incontinence. Furthermore, perioperative care should encompass comprehensive considerations such as hemostasis, infection prevention, and preservation of defecation function. This case provides valuable clinical insights into the one-stage repair of rare, combined perineal multi-organ injuries. Urethral injuries are a prevalent issue in urology, typically categorized as either anterior or posterior. Anterior urethral trauma commonly arises from various sources, such as animal bites, penetrating wounds, foreign object insertion, penile fractures, and medical procedures, with a notable incidence among adolescent individuals. Untimely urethral repair in the acute phase can result in urethral stricture or complete blockage, complicating treatment and significantly impacting patients’ physical and psychological well-being.^[1]^ The anal sphincter complex is shielded anatomically by adipose tissue in the ischiorectal fossa, the gluteus maximus, and the pelvic floor structure. Clinical injuries to this complex are infrequent, primarily occurring during surgical and obstetric procedures, violent trauma to the anal canal, or wartime injuries.^[2]^ However, concurrent injuries to both the urethra and anal sphincter complex due to trauma are uncommon. This study presents a case of combined repair of urethral and anal sphincter complex injuries in a recently admitted patient with specific trauma involving the perineum, sphincter complex, and bulbar urethra. The diagnostic and treatment procedures are detailed below.

## 2. Case report

A 50-year-old male presented at the Emergency Department of the Third People’s Hospital of Longgang District, Shenzhen, at 6:35 pm on April 10, 2025, reporting ongoing bleeding for 40 minutes following a slip injury. The patient described an incident at 5:55 pm, where he slipped and sustained an impalement injury to his perineum from a sharp tree stump, resulting in persistent bleeding and severe perineal pain. Upon admission to the emergency department, he received a provisional diagnosis of “anal and perineal injury.” The patient, of medium build with a body mass index of 19 kg/m^2^, had a prior diagnosis of bronchiectasis but no history of chronic conditions such as coronary heart disease, diabetes, or hypertension. There was no record of prior blood transfusions or surgical procedures, and the patient reported no smoking, alcohol consumption, or other deleterious habits. Furthermore, there was no family history of genetic disorders. Upon admission, the patient presented with a body temperature of 36.5°C, a pulse rate of 105 beats/min, a respiratory rate of 24 breaths/min, and a blood pressure of 140/80 mm Hg. Physical examination revealed no significant abnormalities in the heart, lungs, or abdomen. The liver and spleen were not palpable below the costal margin, and hepatic dullness was within normal limits with no shifting dullness. Renal percussion was non-painful. Normal development was noted in the penis and scrotum without malformations. The prepuce appeared normal without ulcers, tenderness, indurations, or masses. Bilateral inguinal regions showed no redness, swelling, tenderness, or masses.

**Figure 1.**
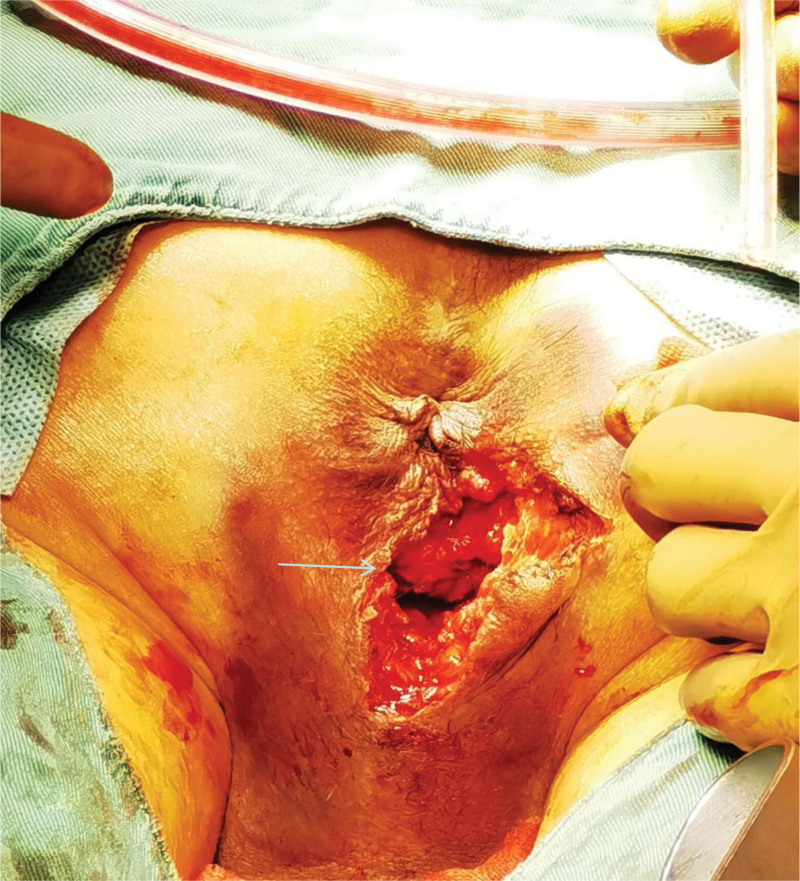
Appearance of perineal injury.

**Figure 2.**
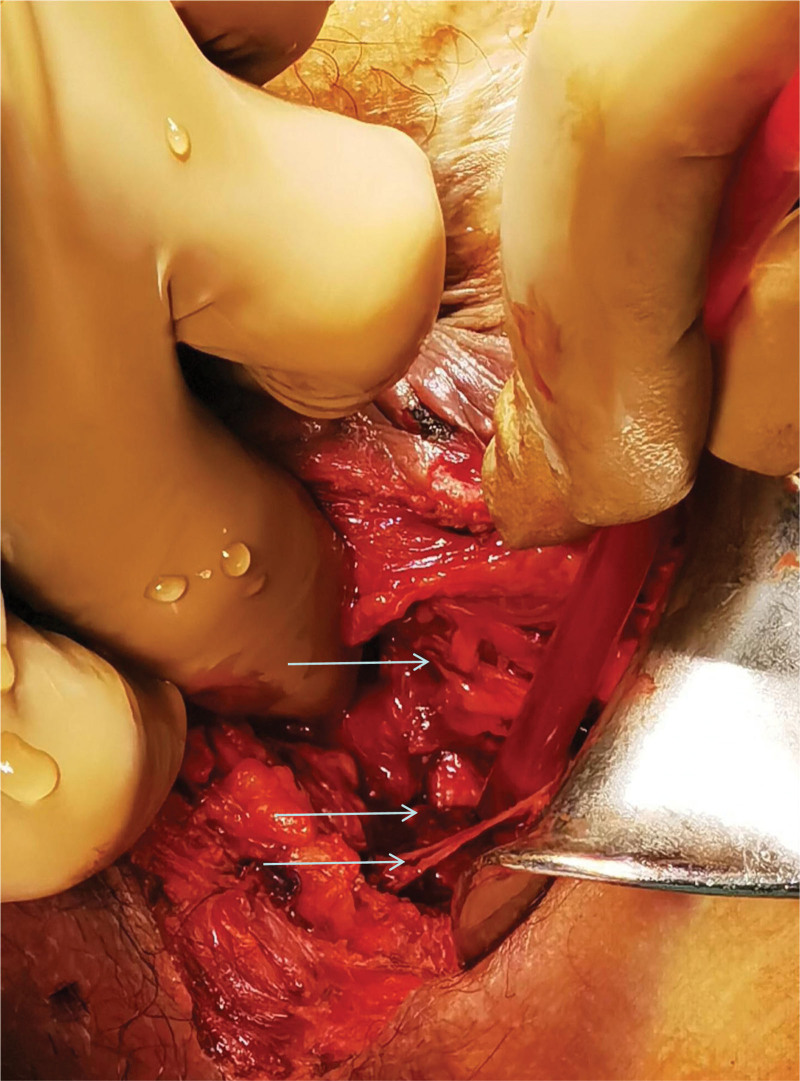
The arrow shows the damaged sphincter complex, urethra and part of the pudendal nerve from top to bottom.

A specialized examination of the anus and perineum revealed an irregular oval wound with everted skin measuring approximately 4 × 3 cm^2^. Subcutaneous hematomas were evident around the wound, on both sides of the scrotum, and at the base of the scrotum. Digital rectal examination indicated intact mucous membranes in the anal canal and rectum with no palpable masses and no blood staining on the finger cot. Exploration of the perineum revealed irregular tears involving the levator ani muscle, external sphincter complex, and superficial and deep transverse perineal muscles. The external wound extended to the puborectal muscle junction and towards the scrotum and levator ani muscle, with a length and depth of approximately 5 and 7 cm, respectively. The wound also extended towards the scrotum and at the 1 o’clock and 3 o’clock positions in the lithotomy position. Approximately 1.5 cm of the urethra was visible at the bulb of the urethra, with lacerations in the surrounding muscles. The urethral mucosa showed thinning and lacerations in some areas (see Figs. 1 and 2).

Auxiliary examinations revealed normal bilateral testicular size and shape on bedside ultrasound, no fluid-filled dark areas in the pelvic and abdominal cavities, no fluid accumulation in the abdominal cavity on abdominal computed tomography, and no pelvic fracture lines or bone fragments. Soft tissue swelling and hematoma were observed in the perineum, with no thickening of the rectal wall. Emergency blood tests showed red blood cell count of 2.57 × 10^12^ g/L (normal range 4.0–5.5 × 10^12^/L), hemoglobin level of 73 g/L (normal range 120–150 g/L), and white blood cell count of 10.2 × 10^9^/L (normal range 3.5–9.5 × 10^9^/L). The diagnosis included urethral laceration, anal sphincter complex injury, perineal injury, and moderate anemia.

## 3. Treatment process

### 3.1. Surgical procedure

**Figure 3.**
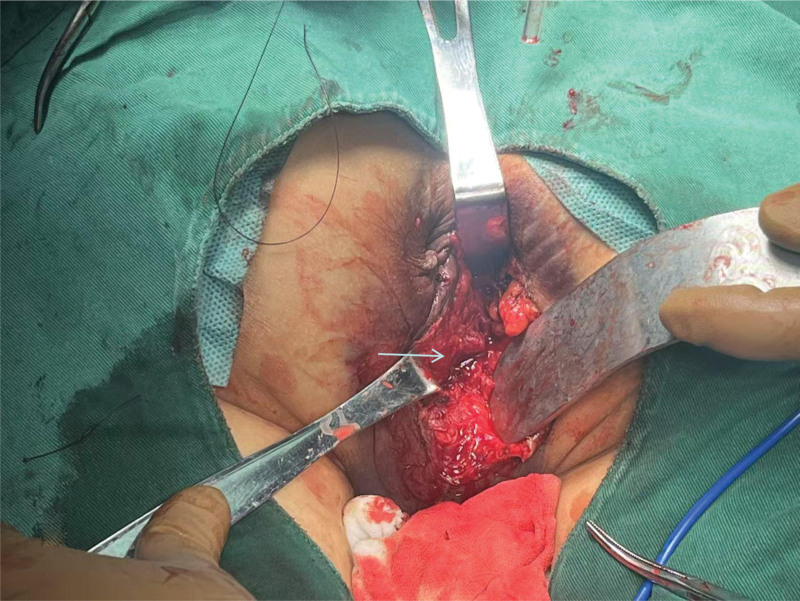
Layer-by-layer suture of the urethral sphincter and sphincter complex.

**Figure 4.**
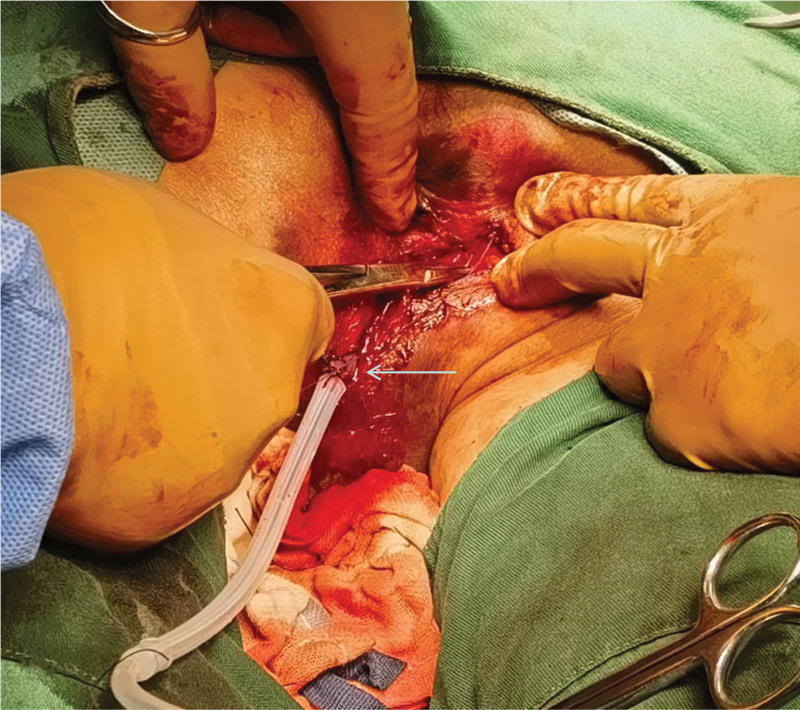
Placement of a drainage tube in the wound.

At 6:55 pm, upon the patient’s hospital admission, intravenous access was established, and same-type packed red blood cells were transfused during fluid replacement. Subsequently, the patient underwent urethral repair, sphincter repair, perineal and anal plasty, and perianal debridement and suture in the operating room under combined intravenous-inhalation general anesthesia with visual laryngoscope intubation. A urinary catheter was inserted preoperatively, yielding light yellow urine, and no bleeding was observed at the external urethral orifice. The patient was positioned prone, revealing intraoperative findings of irregular anal canal injuries with local oozing blood and a significant subcutaneous scrotal hematoma. Exploration unveiled irregular tears in the levator ani muscle, external sphincter complex, and superficial and deep transverse perineal muscles, extending approximately 5 to 7 cm in depth and length towards the scrotum and levator ani muscle, with the deepest point reaching the puborectalis muscle junction. A 1.5-cm segment of the urethra at the bulb of the urethra was visible at the deepest part of the injury, accompanied by irregular lacerations in the external urethral sphincter and urethra, thin urethral mucosa, and intact prostate. Digital rectal examination indicated no intestinal wall communication with the wound surface, and the finger cot remained unstained with blood. Damaged and partially necrotic tissues were excised with tissue scissors, and subcutaneous tissue was incised along the wound surface direction for operative facilitation. The wound surface was cleansed with iodine and hydrogen peroxide cotton balls after meticulous hemostasis, followed by suturing of the lacerated urethral mucosa and external urethral sphincter with 3-Vicryl absorbable sutures. The internal sphincter was intermittently sutured with 2-absorbable sutures, while the external sphincter, superficial, and deep transverse perineal muscles were end-to-end sutured with an overlapping technique using approximately 25 stitches. A negative-pressure drainage tube and urinary catheter were inserted, and the skin edges were aligned and sutured with silk sutures (see Figs. 3 and 4).

### 3.2. Postoperative management

**Figure 5.**
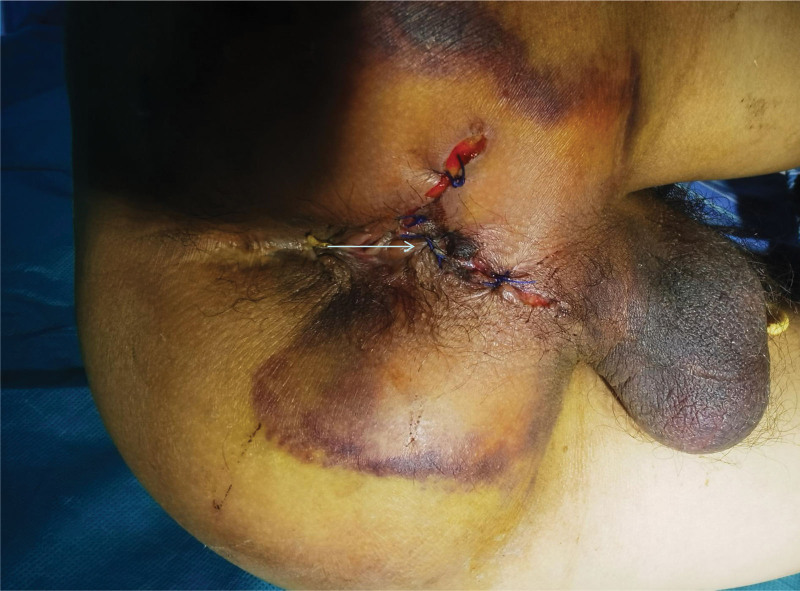
The wound after removing the drainage tube on the 5th day after surgery.

After the operation, the patient was given fasting and local braking for 1 week, and at the same time, intravenous parenteral nutrition was opened to promote wound healing, lactulose oral solution was given on the 7th day after the operation to promote fecal elimination, and a semiliquid diet was given, and blood replenishing drugs were added to treat anemia. Wound treatment included daily iodophor disinfection, hydrogen peroxide cleaning, and potassium permanganate sitz bath after defecation, continuous observation of the suture wound without abnormal redness, swelling, pus, fluid accumulation, etc, and the drainage tube was removed on the 5th day after the operation, when the drainage tube was <1 mL of drainage fluid per day (Fig. 5), and no redness and swelling of the wound was observed, and no oozing of blood and fluid, and no accumulation of fluid was observed, and suture knots started to be removed gradually and intermittently on the 9th day after operation, and fever appeared in the patient on the 20th day after the operation, and the patient had a fever on the 20th day after the operation. On the 20th day after surgery, the patient developed fever and chills, and was considered to have a urinary tract infection, so the urinary catheter was removed, and the patient was cured with antibiotic treatment.

### 3.3. Treatment outcomes

**Figure 6.**
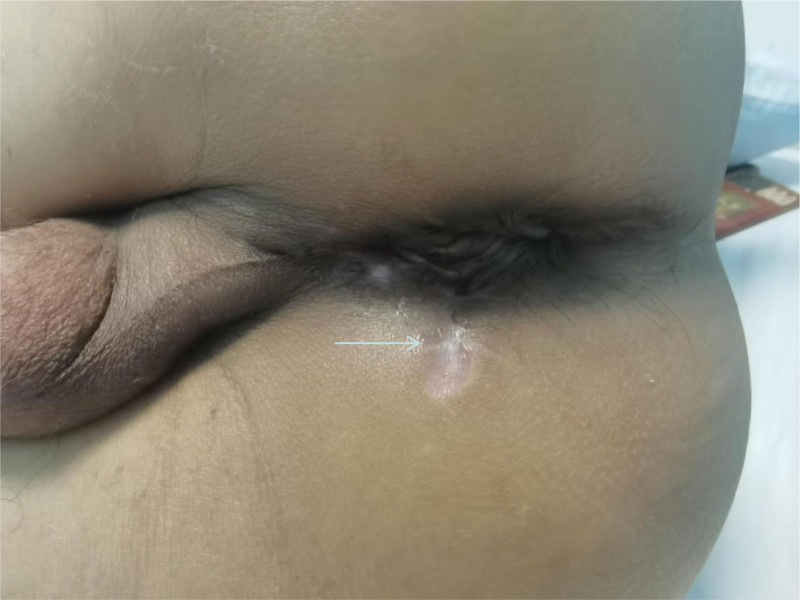
The wound 1 month after surgery.

The patient resumed normal diet on the 7th postoperative day, and at the same time, oral lactulose was taken to keep forming soft stools, and potassium permanganate sitz bath was taken after defecation. The appearance of the anus and perineum returned to normal on the 10th day after the stitches were removed, and the hematoma at the scrotum was gradually absorbed and its appearance was gradually normalized, with no abnormal redness, swelling, pus or fluid accumulation during the period of treatment, and the function of bowel control was normal. The patient was discharged from the hospital on the 10th day after the operation, with smooth urination, no discomfort such as perineal swelling and pain, 2 bowel movements per day, formed stools, and normal bowel control function. One month after the operation, the patient’s follow-up examination: the wound of the perineum was well healed, with no abnormal erythema, pus or fluid accumulation, and there was no suspected or potential abscess or fistula. Anal fingerprinting: the mucosa of the anal canal and rectum was intact, no obvious swelling was detected, and no blood staining was seen when the fingerstick was withdrawn (Fig. 6).

## 4. Discussion

Traumatic injuries to the internal and external anal sphincters and urethra can impact anal fecal control and result in postoperative urethral stricture. Anal fecal control and defecation are vital physiological functions of the anal canal and rectum, reliant on normal anal canal and rectal pressure. Anal canal and rectal pressure encompass resting pressure, maximum systolic pressure, defecation pressure, and rectal sensation. The generation of anal canal resting pressure primarily relies on the contraction activity of the anorectal ring, comprising a circular muscle group formed by the superficial and deep layers of the external anal sphincter, the internal anal sphincter, the levator ani muscle, and the longitudinal muscle of the lower rectum. This complex, known as the anal sphincter complex, encompasses mutually complementary muscles. Damage to this complex, particularly the anal sphincter, can result in fecal incontinence.^[3]^

Anal sphincter injury can result from various factors such as neurological, obstetric, traumatic events, and anal surgery.^[4]^ Surgical repair for patients with anal sphincter complex injury necessitates tailored approaches based on the specific location, depth, and affected muscle groups. For instance, perineal lacerations from childbirth can be categorized by severity for staged treatment involving mucosal repair, sphincter suturing, and superficial and deep transverse muscle reconstruction.^[5,6]^ Research indicates that in cases where damage is limited to less than one-third of the lateral or anterior anal sphincter, simple sphincter repair suffices; however, if the sphincter damage exceeds one-third, anal sphincter reconstruction is advised.^[7,8]^

Repairing trauma or perineal injuries typically involves layer-by-layer restoration of the damaged muscles following anatomical principles. Specifically, repair of internal anal sphincter injury often entails separate suturing, commonly using interrupted or mattress end-to-end sutures, which has been shown to notably decrease postoperative anal incontinence rates.^[9]^ Conversely, external anal sphincter injury repair commonly employs end-to-end or overlapping suture techniques. Studies indicate no significant statistical variance in perineal pain, dyspareunia, fecal incontinence, or overall quality of life between end-to-end and overlapping sutures for the external anal sphincter.^[10]^ Notably, the overlapping suture method significantly reduces postoperative anal incontinence, particularly recommended for complete external anal sphincter ruptures due to its requirement of 2 independent stumps and higher suture tension. During anal sphincter repair procedures, concealing suture knots beneath superficial perineal muscle tissue is advised to mitigate postoperative suture displacement risks.^[11]^ In this instance, a stratified suture approach was utilized, employing the end-to-end suture method for the internal sphincter and the overlapping suture technique for the external sphincter. This methodology is intended to mitigate the incidence of fecal incontinence postoperatively in patients.

Urethral injury is a prevalent condition in urology, with varying treatment approaches among physicians. The initial management of urethral injuries by the primary healthcare provider can significantly influence subsequent treatment and therapeutic outcomes. These injuries are categorized into anterior and posterior types. Anterior urethral injuries typically result from external trauma, such as animal bites, penetrating wounds, foreign body insertion, penile fracture lacerations, and medical procedure-related injuries. Straddle injuries commonly cause testicular trauma. Iatrogenic injuries frequently occur in the penile region, often due to catheterization errors and complications from transurethral procedures. Posterior urethral injuries commonly accompany pelvic fractures, known as pelvic fracture urethral injuries, affecting approximately 10% of pelvic fracture patients.^[12]^ Penetrating injuries to the pelvis, perineum, or buttocks may also involve the posterior urethra, albeit rarely in everyday scenarios, usually in conjunction with other injuries, notably abdominal trauma.^[13,14]^ Female urethral injuries, less frequent than in males, typically result from trauma or iatrogenic causes like childbirth, pelvic fractures, or surgical mishaps. These injuries may predispose individuals to urinary incontinence and urethrovaginal fistula formation.^[15]^

For a patient with an anterior urethral injury, the following options for urethral repair are advised.

### 4.1. Male urethral injury

Closed anterior urethral injuries encompass incomplete anterior urethral injuries and complete anterior urethral ruptures. In cases of incomplete anterior urethral injury, options include catheter insertion,^[16]^ urethral rupture reconstruction, and suprapubic cystostomy. Catheter insertion is suitable for patients with minor partial injuries, allowing for self-repair of the injured urethra. Monitoring the duration of catheter indwelling and post-indwelling urinary tract infections is crucial. If catheter insertion is not feasible, emergency anterior urethral rupture reconstruction can be considered following patient evaluation and consent. In cases where surgery is not well-tolerated or reconstruction is not feasible, suprapubic cystostomy may be performed to drain bladder urine. Complete anterior urethral ruptures, often resulting from trauma, are typically accompanied by extra-urethral tissue injuries leading to local hematoma formation. Urine infiltration into the ruptured end can cause local infection and potential abscess formation if left untreated. Timely urethral rupture reconstruction by an experienced urologist is recommended to ensure successful surgery and patient recovery. Emphasis should be placed on achieving smooth repair and reconstruction of the urethra, along with hemostasis and debridement of the rupture end and injured site to minimize postoperative complications. Open anterior urethral injuries typically result from incidents such as car accidents, falls from heights, or animal bites. A thorough assessment of the injury should include details such as the time, location, cause, and mechanism of the injury, as well as an evaluation of the extent and specific characteristics of the injury. Following a comprehensive assessment, if conditions permit one-stage suture repair of the urethral injury, this approach should be pursued. In cases where the injury is extensive, severe, or the patient’s condition does not allow for immediate one-stage repair, it is advisable to perform suprapubic cystostomy for bladder drainage concurrently with local trauma management. Subsequent to improvement in the patient’s condition, a two-stage urethral reconstruction can be undertaken. Research indicates that providing an accurate and thorough assessment of urethral injuries can effectively reduce the incidence of urethral strictures resulting from such injuries.^[17]^

### 4.2. Female urethral injuries

The current clinical literature on female urethral injuries is limited, primarily comprising case studies and lacking a robust foundation for high-quality clinical research. Presently, one-stage repair is predominantly advocated as the optimal approach for treating female urethral injuries to minimize the risk of post-repair incontinence, discouraging multiple surgeries or two-stage reconstructions. In cases where a patient’s vital signs are unstable or when she is deemed unsuitable for immediate repair surgery due to concurrent complications, it is advisable to place a suprapubic cystostomy tube during stage I and defer the repair procedure.^[13]^ Urethral contusions with intact urethral continuity can be managed through direct indwelling catheterization. Proximal urethral injuries typically necessitate end-to-end anastomosis via the bladder or retropubic route to restore urethral continuity and mitigate the likelihood of urethral fistula, stricture, atresia, and urinary incontinence. For dissections or longitudinal injuries in the mid or distal urethra, reconstructive surgery can be conducted transvaginally. Furthermore, in cases where urethral injuries in women coincide with bladder injuries, concurrent repair of both structures can be carried out through the bladder route.^[16]^

### 4.3. Special urethral injuries

Unique urethral injuries can result from animal bites, wartime trauma, athletic activities, among others. Treatment strategies vary based on the severity of the injury. Mild injuries may be managed with on-site catheterization before transfer to a medical facility, while severe injuries, especially when accompanied by other life-threatening conditions or failed catheterization attempts, typically necessitate open exploration and surgical repair as recommended by the guidelines of the European Association of Urology and the American Urological Association.^[18]^ In cases where evacuation to a medical facility is prolonged, ultrasound-guided suprapubic cystocentesis may be considered. Alternatively, if the patient experiences urinary retention with a palpable bladder, suprapubic cystostomy can be performed to divert urine flow, monitor output, guide fluid management, and facilitate emergency medication administration during transport.

Following stabilization of the patient, the choice of surgical intervention is determined by the location and extent of the urethral injury. Endoscopic urethral commissurotomy remains the preferred in-hospital management approach for specific urethral injuries. This procedure enables early restoration of urethral continuity or shortening of the injury length, thereby reducing the risk of urethral deformities and stenosis.^[19]^ Notably, Chapple et al^[20]^ suggested that penile urethral defects up to 1.5 cm and bulbous urethral defects up to 2 cm can be effectively repaired through one-stage surgery, while larger defects are best addressed through two-stage urethral reconstruction. In cases of posterior urethral injuries concurrent with fractures and other urgent surgical needs, guidelines recommend considering urethral commissurotomy during the initial surgery to lower the risk of complications such as urethral strictures post one-stage repair. Subsequent decisions regarding additional endoscopic interventions or delayed urethral repair surgery are based on voiding patterns and urethral paracentesis outcomes.

In conclusion, optimal treatment strategies for patients with such trauma should encompass the following key elements: thorough preoperative assessment, including detailed evaluation of injury specifics (time, location, cause, and site), consideration of inter-tissue relationships, determination of ideal surgical timing, and comprehensive assessment of cardiovascular, respiratory, and overall health status. Identification of potential risk factors impacting surgical outcomes and patient safety is crucial. Profound understanding of anatomical intricacies within the surgical region, particularly in procedures involving the anus, perineum, and urethra, which entail intricate networks of muscles, nerves, and blood vessels. Proficiency in anatomical relationships aids in safeguarding vital tissues, promoting functional recuperation, and mitigating postoperative complications. Selection of suitable sutures and adept application of surgical techniques to prevent wound dehiscence, a common cause of surgical repair failure. Traditional surgeries often utilize absorbable sutures; however, inadequate tightening by the surgeon coupled with postoperative patient movement can lead to suture loosening or breakage, resulting in wound dehiscence and surgical inadequacy. Emphasis on meticulous postoperative care to enhance recovery outcomes. Measures to prevent defecation straining within the initial 7 days post-surgery are imperative, necessitating controlled diet, stool softeners, infection prevention protocols, and enhanced perineal hygiene and care.

## 5. Limitation

While this detailed case analysis provides important clinical insights, we acknowledge the need for larger prospective studies. In the future, large-sample and multicenter studies can be conducted.

## Acknowledgments

We would also like to express our gratitude to all the researchers who participated in this study, including doctors, nurses, as well as the editors and reviewers who provided suggestions for the revision of this article.

## Author contributions

**Conceptualization:** Jingxing Chen, Xiao Wang.

**Data curation:** Jingxing Chen, Xiao Wang.

**Formal analysis:** Jingxing Chen.

**Funding acquisition:** Jingxing Chen.

**Investigation:** Jingxing Chen, Qian Liang.

**Methodology:** Jingxing Chen, Qian Liang.

**Project administration:** Jingxing Chen.

**Resources:** Jingxing Chen, Wei Peng.

**Software:** Jingxing Chen, Wei Peng.

**Supervision:** Jingxing Chen.

**Validation:** Jingxing Chen, Ningchao Du.

**Visualization:** Jingxing Chen.

**Writing – original draft:** Jingxing Chen, Ningchao Du.

**Writing – review & editing:** Ningchao Du.
